# Evaluation of broiler meat in experimental listeriosis

**DOI:** 10.5455/javar.2022.i580

**Published:** 2022-03-13

**Authors:** Iryna Borovuk, Nadiia Zazharska

**Affiliations:** Faculty of Veterinary Medicine, Dnipro State Agrarian and Economic University, Dnipro, Ukraine

**Keywords:** Broiler chickens, *Listeria *spp., meat storage, total plate count, physicochemical indicators

## Abstract

**Objective:**

The work aimed to assess the safety and quality of broiler meat in experimental listeriosis changes in storage.

**Materials and Methods:**

Ross Cobb 500 chickens (40) were divided into 4 groups of 10 animals each. Chickens from three experimental groups were infected by *Listeria innocua*,* Listeria ivanovii*, and* Listeria monocytogenes*. Meat samples were stored for 5 days at 0°C–4°C. Meat samples were kept in the refrigerator for 3, 4, and 5 days. Microbiological and laboratory indicators of meat freshness were found on these days as well.

**Results:**

After the slaughter of chickens with experimental listeriosis, pathological changes in muscles and organs were noted against the background of fattening carcasses with a high slaughter yield. By bacterial contamination, 1 day after slaughter, the meat of chickens of the experimental groups (*L. innocua*, *L. ivanovii*, and *L. monocytogenes*) outperformed the control group by almost 1.9, 13.9, and 24.7 times, respectively (*p* < 0.05). The same trend is observed for the third, fourth, and fifth days of meat storage. To keep chicken meat fresh for 5 days, only samples from the control group stayed fresh.

**Conclusion:**

According to the total bacterial contamination, the meat of chickens of the groups *L. innocua *and *L. ivanovii* was dangerous to human health after 5 and 4 days of storage, respectively. From the first day after the chickens were killed, the meat of chickens that had been infected with *L. monocytogenes* did not meet the requirements (up to 100 CFU/gm) and was not safe to store or eat.

## Introduction

Nowadays, food safety is a cornerstone for the food industry and the poultry industry [1]. Evans et al. [2] noted that in addition to an effective food safety management system, the perceptions of risk, control, and responsibility within a food manufacturing business are essential and influential factors associated with managing Listeria monocytogenes. Recently, scientists have drawn attention to listeriosis’s growing importance as a foodborne disease. Among the various species, L. monocytogenes is the most commonly reported causative agent of listeriosis in both humans and birds. The standard method of human infection is by consuming foods contaminated with L. monocytogenes [3–7].

The study by Bechtel and Gibbons [8] aimed to determine if there was an association between genetically different populations of L. monocytogenes and certain foods, especially cheese and other dairy products. It is proven that counter-sliced turkey delicatessens can be a reservoir for L. monocytogenes growth and cross-contamination [9]. The research material of Mcminn et al. [10] was chicken patties, chicken tenders, beef patties, and frankfurter batter. Many scientists are preoccupied with the tolerance of L. monocytogenes to biocides [11–16]. Other scientists are also studying the effects of various substances on biofilms of L. monocytogenes [17,18]. Hua et al. [19] examined the efficacy of the pilot blancher for steaming to kill *Listeria* innocua and L. monocytogenes on surfaces, including surfaces in food processing plants in contact with food. Palaiodimou et al. [20] are concerned with the genomes of different Listeria species found in food processing plants. The goal of the study was to look at the safety and quality of broiler meat in an experiment with listeriosis and changes in how it was stored.

## Material and Methods

Execution of experimental research on broiler chickens was accompanied by observance of all bioethical requirements according to the requirements of the European Convention for the Protection of Vertebrate Animals Used for Experimental and Other Scientific Purposes and complied with the Law of Ukraine “Law of Ukraine” “On the Protection of Animals from Cruelty.”

### Experiment scheme

Ross Cobb 500 chickens (n = 40) at the age of 10 days, weighing an average of 240 gm, were divided into 4 groups of 10 animals. Broilers were kept in cages on the same diet and in identical conditions, and chickens had free access to food and water. The first group was a control, the second group was infected with L. innocua, the third group was Listeria ivanovii, and the fourth group was infected with L. monocytogenes. The infection was performed orally on the 15th day of life with a daily culture at a 0.5 Mac Farland-1 ml dose, which was 1.5 × 108 CFU/cm3. For infection of chickens, we used reference cultures of microorganisms purchased from the State Research and Control Institute of Biotechnology and strains of microorganisms indigenous to Ukraine, namely: L. monocytogenes UNCSM–041, L. ivanovii UNCSM–042, and L. innocua UNCSM–043.

The slaughter took place on the 38th day of the broiler’s life. During the bleeding of chickens, blood samples were taken for biochemical and hematological studies. The carcasses were cooled to 2°C. It was found out how much poultry carcass yield, the weight of parenchymal organs (liver, heart, stomach, and fillets), and how much meat was in each piece.

To study the quality and safety of the meat of broiler chickens in experimental listeriosis, organoleptic, physicochemical, and microbiological parameters were determined. Meat samples were placed in plastic bags and stored for 5 days at a temperature of 0°C–4°C.

### Analysis

Organoleptic and laboratory tests were performed in the bacteriological and chemical-toxicological departments of the Dnipropetrovsk Regional State Laboratory of the State Service of Ukraine on Food Safety and Consumer Protection. Biochemical and hematological studies of chicken blood were performed at the biosafety center of Dnipro State Agrarian and Economic University. During the organoleptic evaluation of carcasses, attention was paid to how the carcasses looked, if there were any pathological changes or fat, how the color, smell, and muscle elasticity looked and felt.

The broth was evaluated for organoleptic properties (aroma, color, and transparency). Microbiological and physicochemical studies of meat were performed on the day of slaughter and the third, fourth, and fifth days of storage of meat samples. On the first day of meat storage, the water-holding capacity of meat, moisture, protein, fat, and pH were also determined. On the third, fourth, and fifth days of storage, the content of ammonia and ammonium salts, volatile fatty acids, pH, products of the primary breakdown of protein, acid, and peroxide value of chicken fat were determined. 

Water-holding capacity is determined by Fernandes et al. [27]. Meat moisture was determined using a weight or gravimetric method. The protein was found using a technique called Kjeldahl, which is based on the mineralization of the sample. Ammonia was distilled into a sulfuric acid solution, and then the test sample was titrated. 

The extraction method performs the determination of the fat content of meat. Microbiological studies of poultry meat (total plate count, coliforms, *Salmonella*, number of Listeria) were conducted. The pectoral and thigh muscles were selected for microbial examination. For microscopy, the smears were Gram-stained in a Hooker modification. 

Plate count agar (HiMedia) was used to determine the total plate count. Plates with inoculations were placed on a thermostat at 30°C. After 72 h, an automatic colony counter Scan-500, was used to count colonies.

Detection of coliform bacteria was carried out using violet-red bile agar, in accordance with ISO 4832 (2006), and incubated for 48 h at 37°C.

Isolation of *Salmonella* was performed according to ISO 6579-1: 2017 using two selective enrichment media Modified Rappaport Vassiliadis Medium and Mueller Kauffman Tetrathionate Broth Base and two differential diagnostic media, Xylose Lysine Deoxycholate Agar and *Salmonella* Differential Agar Modified.

Detection and counting of *Listeria* were carried out in accordance with CSN ES ISO 11290-2: 2017 [21]. Isolation of *Listeria* was performed using Fraser broth base (HiMedia), inoculation on differential diagnostic media—Oxford agar and agar-ALOA (HiMedia). Inoculations of microorganisms were kept in a thermostat at a temperature of 37°C ± 1°C for 24–48 h.

Using steam distillation, volatile fatty acids were isolated, and their content was determined by titration of potassium hydroxide with phenolphthalein indicator until the crimson color faded away to the point where it was no longer visible.

Detection of ammonia and ammonium salts illustrates the accumulation of ammonia in muscle and the breakdown of protein (meat spoilage) using Nessler’s reagent, which forms a complex salt with ammonia iodide dimercuramonium transparent yellow (orange) color. If the extract has a greenish-yellow color and is still clear, the meat is fresh. If the extract has a bright orange color with bubbles and sediment, the meat is stale and not very fresh at all.

To determine the products of the primary breakdown of protein in the broth, copper sulfate was added. Copper sulfate precipitates the products of enzymatic hydrolysis of proteins that have accumulated during the decomposition of the meat and passed into the broth during the cooking of the sample. The broth does not change color and remains transparent (like fresh meat). The meat will be of dubious freshness if there is slight turbidity in the broth. In the presence of protein breakdown, flakes, turbidity, and a greenish color are formed (stale meat). For potentiometric measurement, use a pH meter with pH-150 MH.

The acid value of fat is the number of milligrams of caustic KOH or NaOH required to neutralize the free fatty acids contained in 1 gm of fat. The determination method is based on neutralizing an alcohol-benzene solution (1:1) of fat with NaOH or KOH. This is the number of grams of iodine that can be taken from potassium iodide by peroxides in 100 gm of fat.

### Statistical analysis

Microsoft Exel and Statisica 12 (Stat Soft) softwares analyzed the data. The data in the tables are presented as *x* ± SE (*x* ± standard error). Differences between values in the groups were determined using the Tukey test, where the differences were considered to be significant at *p* < 0.05 (subject to the Bonferroni Amendment).

## Results

At the end of the experiment, in the control group of chickens, all animals (*n* = 10) were alive; in the groups of *L. innocua*, *L. ivanovii*, *L. monocytogenes* left 8, 9, and 7 birds, respectively.

Even though some chickens died in the infected groups, other animals slightly outnumbered the group, trying to keep their weight down.

According to the biochemical and hematological studies of the blood of chickens in the control and experimental groups, almost all indicators did not differ statistically. The creatinine in the blood of broiler chickens in the groups of *L. innocua* and *L. ivanovii* was lower than the control by 14.9% and 13.6%, respectively (*p* < 0.05).

### Inspection of meat

The skin and visible mucous membranes remain unchanged when inspecting broilers before slaughter, and the crest is red. The slaughter of chickens took place in compliance with sanitary and hygienic requirements. During the slaughter inspection, the fatness of broiler carcasses of all groups was noted.

The carcasses in the control group had well-developed pink muscle tissue. In the cavity, the serous membranes were shiny and moist. The smell was typical of fresh meat, and fat is pale yellow, homogeneous, and has a characteristic fresh odor.

**Figure 1.**
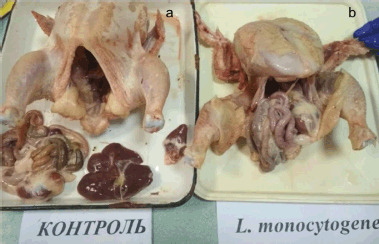
Single dotted and finely spotted hemorrhages in the subcutaneous tissue on the wings of a broiler infected with *L. monocytogenes.* (a) A carcass from the control group. (b) A carcass from a group infected with *L. monocytogenes*.

During the post-mortem veterinary and sanitary examination, significant hemorrhages, congestive venous hyperemia, and signs of damage to specific organs in the experimental groups of broilers were revealed. *Listeria monocytogenes* chicken carcasses showed poor bleeding, like bleeding under the skin in the wing area (Fig. 1).

**Figure 2.**
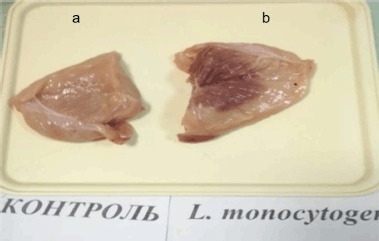
Hemorrhagic infiltration of pectoral muscles, infiltration. (a) Chicken fillet from the control group. (b) Chicken fillet from the group infected with *L. monocytogenes*.

In a group of chickens that had been infected with *L. monocytogenes*, blood was found in the pectoral muscles (Fig. 2).

**Figure 3.**
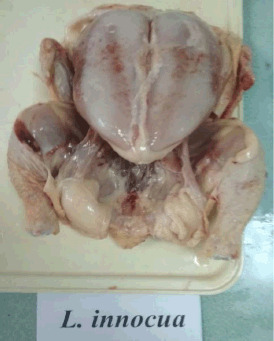
Carcass of poultry infected with *L. innocua.* Multiple hemorrhages in the subcutaneous tissue.

**Figure 4.**
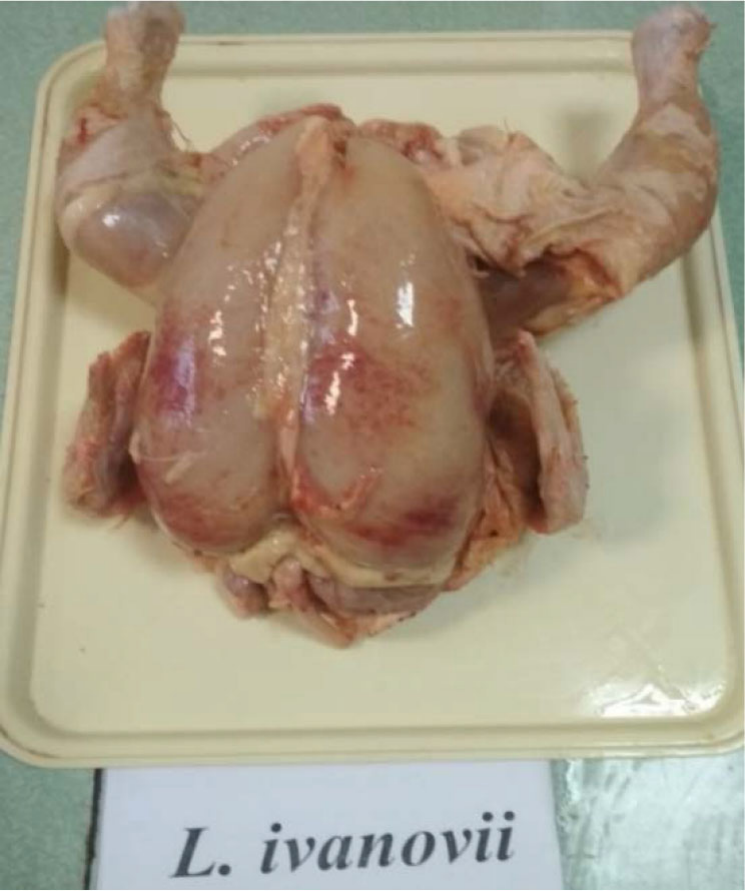
Carcass of poultry infected with *L. ivanovii*. Spotted multiple hemorrhages in the subcutaneous tissue.

There were hemorrhages in the subcutaneous tissue of the carcasses of *L. innocua* and *L. ivanovii* groups in the pictures. The fatness of the carcasses made them look bad (Figs. 3 and 4).

**Figure 5.**
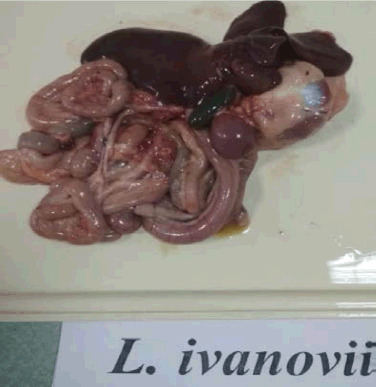
Congestive hyperemia of internal organs from the carcass of a broiler infected with *L. ivanovii*. Intestinal vascular hyperplasia.

During the post-mortem examination of *L. ivanovii* chickens, an enlargement of the spleen, an overfilled gallbladder, congestive hyperemia of the internal organs, and hyperplasia of the intestinal vessels were observed (Fig. 5).

Meat broth was monitored by tasting. The broth of the control group of broiler chickens received the highest number of points. Fragrant, rich, transparent. The meat broth from *L. innocua* and* L. ivanovii* was less fragrant than the broth from the control group and had a grayish tinge. However, the amount of fat was almost the same, which indicates the fatness of the bird regardless of the carrier of the pathogen. The broth in the group of chickens *L. monocytogenes* generally had no aroma. *Listeria*-infected chickens weighed about the same as chickens in the control group. This shows that *Listeria* does not affect the growth and development of broilers (Table 1).

By weight, the chickens of the control and experimental groups were almost at the same level. Broilers from the groups L. innocua and L. ivanovii slightly outperformed the control group (by 1.6% and 4.4%, respectively). By carcass weight, the broilers of the experimental groups slightly beat the control group (L. innocua, L. ivanovii, and L. monocytogenes; 1.0%, 5.0%, and 3.6%, respectively), but a statistical difference was not found. The broilers in the experimental group are a little heavier because they have more moisture in their meat.

Carcass yield of broiler chickens in all groups was observed at almost the same level, 76.4%–77.3%. If you put *L. ivanovii*, *L. monocytogenes*, and *L. innocua* in a chicken, its liver weight grew by 11.6% and 13.4%. There was no statistical difference, and no statistical difference was found by 9.3% and 13.2%, respectively, compared to the control group. By stomach weight, chickens of the L. ivanovii group were almost on the same level as the control group, and in the L. innocua group, by 6.6% more. The largest stomach weight was found in the group of chickens infected with L. monocytogenes, 27.5% more than the control group of poultry.

**Table 1.** Indicators of weight of carcasses and offal of broiler chickens, (*x* ± SE, *n* = 7).

Indicator	Group			
	Control	*L. innocua*	*L. ivanovii*	*L. monocytogenes*
Weight of the poultry, gm	2,320.0 ± 70.8	2,358.0 ± 68.9	2,422.8 ± 63.4	2,278.5 ± 169.6
Carcass weight, gm	1,783.9 ± 53.6	1,801.5 ± 56.1	1,872.8 ± 55.2	1,847.7 ± 121.9
Carcass yield, %	76.93 ± 0.68	76.38 ± 0.47	77.27 ± 0.62	76.91 ± 0.99
Liver weight, gm	54.3 ± 2.2	60.6 ± 3.9	61.6 ± 5.3	60.6 ± 2.8
Heart weight, gm	12.9 ± 0.7	14.1 ± 1.0	14.1 ± 1.1	14.6 ± 0.7
Stomach weight, gm	25.8 ± 1.4	27.5 ± 1.0	25.7 ± 2.3	32.9 ± 3.2
Fillet weight, gm	534.4 ± 19.5	501.3 ± 22.7	545.6 ± 17.9	493.0 ± 43.0

**Table 2.** Physico-chemical parameters of broiler chicken meat after slaughter, (*x* ± SE, *n* = 7).

Indicator	Group			
	Control	*L. innocua*	*L. ivanovii*	*L. monocytogenes*
Water-holding capacity, %	69.2 ± 0.7	69.5 ± 0.9	69.5 ± 1.0	67.0 ± 1.3
Moisture, %	73.23 ± 0.4^a^	75.99 ± 0.4^b^	74.69 ± 0.2^b^	74.73 ± 0.2^b^
Protein, %	21.50 ± 0.2^a^	20.31 ± 0.4^ab^	20.41 ± 0.1^b^	21.30 ± 0.2^a^
Fat, %	4.49 ± 0.2^a^	4.09 ± 0.4^ab^	4.43 ± 0.2^a^	3.30 ± 0.3^b^

The lowest weight of fillets, the most valuable part of the broiler, was found in chickens of L. monocytogenes and L. innocua, which were 7.8% and 6.2%, respectively, less than the control indicator. The fillet weight of L. ivanovii chickens was 2.1% higher than in the control group, but no statistical difference was found.

### Physico-chemical composition of meat

The water-holding capacity of meat did not differ significantly in groups of broiler chickens infected with Listeria spp. (Table 2). This indicator in the groups of broiler chickens infected with L. innocua and L. ivanovii was at the same level, 0.3% more than in the control group. In the group of L. monocytogenes, the water-holding capacity of meat was lower by 2.2% than the control indicator, but no statistically significant difference was found. The experimental infection of broiler chickens with *Listeria* did not change the ability of poultry meat to hold water.

The moisture in the meat of the control group of broiler chickens was 73.23% and infected with the listeriosis pathogen groups L. innocua, L. *ivanovii,* and L. *monocytogenes,* which was increased by 2.76%, 1.46%, and 1.50%, respectively (p < 0.05).

The protein content of the meat of broiler chickens of the L. ivanovii group was less than that of the control and L. monocytogenes groups by 1.09% and 0.89%, respectively (p < 0.05).

Different letters indicate selections that significantly (*p* < 0.05) within the line differ from each other according to the results of the Tukey test, with Bonferroni correction; if there are no letters above the numbers in the line, then no significant difference between any selections is registered.

The fat content of the meat of chickens of the group L. monocytogenes was less than that of the group of L. ivanovii and control by 1.13% and 1.19%, respectively. A statistical difference was found (p < 0.05). According to organoleptic parameters, broiler meat on the third day of storage corresponded to fresh. Poultry meat from infected groups had a sour smell on the fourth and fifth days. The fat had a slightly grayish tinge.

During the microbiological control of meat safety after slaughter, the experimental samples did not reveal: *Salmonella, Pseudomonas, Proteus, Morganella, Providencia, Campylobacter, Staphylococcus aureus, Streptococcus, Clostridium perfringens, Bacillus cereus, Yersinia, *Enterobacteriaceae, Enterococcus, and Escherichia *сoli.* The study of coliforms was performed once, after slaughter. No coliforms were detected in the L. innocua and control groups. Bacteria from coliforms were found in three and four samples from the groups *L. ivanovii* and *L. monocytogenes*, but not for the other two.

**Table 3.** Bacterial contamination and *Listeria *spp. in the meat of broilers, (*x* ± SE, *n* = 7).

Indicator	Shelf life of meat after slaughter, day	Group			
		Control	*L. innocua*	*L. ivanovii*	*L. monocytogenes*
Total plate count, ×10^3^ CFU/gm	1	0.1 ± 0.005^a^	0.3 ± 0.017^b^	1.9 ± 0.034^c^	3.4 ± 0.075^d^
	3	1.1 ± 0.052^a^	2.5 ± 0.193^b^	3.0 ± 0.075^b^	4.0 ± 0.186^c^
	4	1.5 ± 0.053^a^	5.5 ± 0.436^b^	13.1 ± 0.800^c^	33.3 ± 1.017^d^
	5	1.7 ± 0.049^a^	51.1 ± 2.632^b^	29.1 ± 0.911^c^	144.3 ± 7.825^d^
*Listeria *spp., CFU/gm	1	0.0 ± 0.0^a^	56.0 ± 1.7^b^	83.7 ± 1.9^c^	109.6 ± 3.3^d^
	3	0.0 ± 0.0^a^	78.6 ± 2.7^ b^	157.1 ± 9.2^ c^	242.9 ± 6.1^ d^
	4	0.0 ± 0.0^a^	142.9 ± 4.7^ a^	265.7 ± 3.7^ b^	315.7 ± 5.7^ c^
	5	0.0 ± 0.0^a^	261.4 ± 3.4^a^	320.0 ± 8.2^b^	411.4 ± 3.4^ c^

### Microbiological indicators of meat

After the slaughter of broilers, cultures (L. innocua, L. ivanovii, and L. monocytogenes) were isolated in the respective groups. In the meat of the control group of broiler chickens, microorganisms of the genus Listeria were not detected. Bacterial contamination and Listeria CFU/gm were carefully monitored during the storage of meat of different groups – 1, 3, 4, and 5 days after slaughter (Table 3).

Bacterial contamination of carcasses after slaughter corresponded to the permissible level of contamination of chilled meat of broiler chickens—10^4^ CFU/gm according to the permissible level adopted in Ukraine. However, the total plate count of chicken meat samples from the experimental groups of L. innocua, L. ivanovii, and L. monocytogenes compared to the control group was almost 1.9, 13.9, and 24.7 times higher, respectively (p < 0.05).

Different letters indicate selections that significantly (*p* < 0.05) within the line differ from each other according to the results of the Tukey test, with Bonferroni correction; if there are no letters above the numbers in the line, then no significant difference between any selections is registered.

On the third day of storage of chicken meat in all groups, the level of bacterial contamination had a rapid tendency to increase, but was within acceptable limits (up to 10^4^ CFU/gm). Compared with the control group, the total plate count of chicken meat samples from the groups L. innocua, L. *ivanovii, *L. monocytogenes was 2.2, 2.7, and 3.6 times higher (p < 0.05), respectively. Moreover, this indicator in the group of L. monocytogenes was statistically higher compared to other experimental groups (p < 0.05).

On the fourth day after slaughter, the number of microorganisms in the meat increased significantly. Bacterial contamination of the meat of chickens of the groups L. ivanovii and L. monocytogenes exceeded the permissible level of contamination (up to 10^4^ CFU/gm), which indicated spoilage of the meat. The total plate count of meat of groups L. innocua, L. ivanovii, and L. monocytogenes was 3.8, 9.0, and 22.8 times higher than in the control group, respectively (p < 0.05).

On the fifth day of storage, the level of bacterial contamination of the meat of chickens infected with Listeria was significantly higher than the permissible value for chilled meat (10^4^ CFU/gm). Compared with the control group, the total plate count of chicken meat of the L. innocua, L. ivanovii, and L. monocytogenes groups was 30.1, 17.1, and 84.9 times higher, respectively (p < 0.05).

The maximum shelf life of chilled poultry meat is 5 days. Only samples from the control group remained fresh in terms of organoleptic parameters. According to the results of the research, on the fourth and fifth day of storage, the meat of chickens infected with Listeria was dangerous to human health.

According to Commission Regulation (EC) No. 2073/2005 [22] on microbiological criteria for foodstuffs, the level of contamination of L. monocytogenes is essential for the safety of poultry meat. The food market operator must guarantee to the consumer the amount of L. monocytogenes not more than 100 CFU/gm until the end of the shelf life of the product (5 days). From the first day of storage until the end of the shelf life, the amount of L. monocytogenes in the meat of chickens infected with this pathogen exceeded the maximum allowable level. Listeria is a psychrotrophic microorganism capable of growing at temperatures below 0°C. Unlike most pathogenic microorganisms, the temperature of the refrigerator (0°C–4°C) contributes to the preservation and slow reproduction of *Listeria*.

*Listeria* spp. was not isolated from meat samples of the control group during the experiment. After the slaughter in chickens infected with L. innocua, the number of CFU of listeriosis pathogens was 1.5 and 2 times less than the group of L. ivanovii and L. monocytogenes, respectively (p < 0.05).

On the third day of storage, the number of *Listeria* in the meat of L. innocua chickens was two and three times less than in the group of L. ivanovii and L. monocytogenes, respectively (p < 0.05).

**Table 4.** pH level in the meat of broiler chickens (*x* ± SE, *n* = 7).

Shelf life of meat after slaughter, day	Group			
	Control	*L. innocua*	*L. ivanovii*	*L. monocytogenes*
1	6.02 ± 0.04	5.99 ± 0.03	6.11 ± 0.04	6.05 ± 0.03
3	6.19 ± 0.03	6.08 ± 0.03	6.15 ± 0.03	6.13 ± 0.03
4	6.33 ± 0.03^a^	6.34 ± 0.05^ab^	6.56 ± 0.05^b^	6.44 ± 0.09^ab^
5	6.66 ± 0.07^a^	6.71 ± 0.03^a^	6.81 ± 0.03^a^	7.16 ± 0.06^b^

**Table 5.** The content of volatile fatty acids in the meat of broiler chickens, mg KOH/100 gm (*x* ± SE. *n* = 7).

Shelf life of meat after slaughter, day	Group			
	Control	*L. innocua*	*L. ivanovii*	*L. monocytogenes*
3	3.99 ± 0.12	4.47 ± 0.24	4.61 ± 0.35	4.37 ± 0.23
4	4.24 ± 0.09^a^	5.51 ± 0.33^b^	5.49 ± 0.36^ b^	6.00 ± 0.30^b^
5	4.50 ± 0.10^a^	9.17 ± 0.07^b^	9.23 ± 0.07^b^	10.06 ± 0.25^ с^

On the fourth day, the number of Listeria in the meat of chickens of the L. ivanovii and L. monocytogenes groups was 1.9 and 2.2 times higher, respectively, compared with the L. innocua group (p < 0.05). On the fifth day of storage, the number of Listeria in the meat of chickens of groups L. ivanovii and L. monocytogenes was 1.2 and 1.6 times higher, respectively, compared with group L. innocua (p < 0.05).

Therefore, from the first day after slaughter, the meat of broiler chickens with experimental infection with L. monocytogenes by contamination with this pathogen did not meet the requirements and was not suitable for storage and consumption. According to total plate count, the meat of broiler chickens with experimental infection with L. innocua and L. ivanovii was dangerous to human health after 5 and 4 days of storage, respectively.

### Changes in meat during the storage

Throughout the shelf life of broiler meat, the pH level of all samples gradually increased (Table 4). After slaughter, the pH of the meat did not differ statistically between the control and experimental groups. On the third day of storage, the pH of the meat of chickens of all groups was almost at the same level. On the fourth day, the pH level of the meat of chickens in the control group was lower than that of L. ivanovii. A statistical difference was found (p < 0.05). On the fifth day of storage, the pH of the meat of chickens of the L. monocytogenes group was statistically higher compared to the control group, L. innocua, and L. ivanovii by 7.5%, 6.7%, and 5.1%, respectively (p < 0.05).

Different letters indicate selections that significantly (*p* < 0.05) within the line differ from each other according to the results of the Tukey test, with Bonferroni correction; if there are no letters above the numbers in the line, then no significant difference between any selections is registered.

Detection of volatile fatty acids in the meat of broiler chickens was performed starting from the third day (Table 5). The content of volatile fatty acids indirectly reflects the intensity of the oxidation of meat fats in samples. The level of volatile fatty acids up to 4.5 is allowed in fresh meat, 4.5–9 is in dubious fresh meat, and more than 9 mg KOH/100 gm is in stale meat of broiler chickens.

On the third day of storage of the samples, the amount of volatile fatty acids in the meat of chickens of the experimental groups increased by 9.5%–15.5% compared to the control, but no statistical difference was found.

Different letters indicate selections that significantly (*p* < 0.05) within the line differ from each other according to the results of the Tukey test, with Bonferroni correction; if there are no letters above the numbers in the line, then no significant difference between any selections is registered.

On the fourth day of storage, the amount of volatile fatty acids increased rapidly in the meat of poultry infected with *L. innocua, L. ivanovii, *and *L. monocytogenes*, compared with the control group by 30.0%, 29.5%, and 41.5%, respectively (*p* < 0.05). The data obtained show that chickens infected with *Listeria* spoils faster than the meat of chickens in the control group.

On the last fifth day of storage, this indicator showed a significant increase in broiler meat in all infected groups of *L. innocua, L. ivanovii, *and *L. monocytogenes*—2–2.2 times the control group (*p* < 0.05). Moreover, this indicator in the group of *L. monocytogenes* was statistically higher compared to other experimental groups (*p* < 0.05). The color of the extract was used to figure out how much ammonia and ammonium salts were in the sample. The samples were put into three groups: fresh, dubious fresh, and stale (Table 6).

**Table 6.** Samples of broiler chicken meat by ammonia and ammonium salts (*n* = 7).

Shelf life of meat after slaughter, day	Group. meat fresh/dubious fresh/stale			
	Control	*L. innocua*	*L. ivanovii*	*L. monocytogenes*
3	7/0/0	4/3/0	4/3/0	3/4/0
4	7/0/0	1/2/4	0/3/4	1/0/6
5	6/1/0	0/0/7	0/0/7	0/0/7

**Table 7.** Samples of broiler chicken meat by reaction with CuSO_4_ (determination of the products of the primary breakdown of protein), (*n* = 7).

Shelf life of meat after slaughter, day	Group. meat fresh/dubious fresh/stale			
	Control	*L. innocua*	*L. ivanovii*	*L. monocytogenes*
3	7/0/0	7/0/0	7/0/0	7/0/0
4	7/0/0	1/2/4	0/3/4	1/0/6
5	6/1/0	0/0/7	0/0/7	0/0/7

**Table 8.** Peroxide and acid value of fat, (*x* ± SE, *n* = 7).

Indicator	Shelf life of meat after slaughter, day	Group			
		Control	*L. innocua*	*L. ivanovii*	*L. monocytogenes*
Acid value, mg KOH/gm	3	0.55 ± 0.02^a^	0.98 ± 0.18^ab^	1.15 ± 0.17^b^	1.17 ± 0.12^b^
	4	0.84 ± 0.05^a^	1.73 ± 0.20^b^	2.01 ± 0.25^b^	2.35 ± 0.21^b^
	5	0.98 ± 0.07^a^	2.60 ± 0.03^b^	2.57 ± 0.05^b^	2.65 ± 0.04^b^
Peroxide value, mg I_2_/100 gm	3	0.008 ± 0.001^a^	0.019 ± 0.002^b^	0.015 ± 0.003^ab^	0.020 ± 0.001^b^
	4	0.009 ± 0.001^a^	0.034 ± 0.005^b^	0.037 ± 0.005^b^	0.044 ± 0.005^b^
	5	0.013± 0.003^a^	0.045 ± 0.002^b^	0.044 ± 0.003^b^	0.056 ± 0.004^b^

On the third day in the control group, all samples were fresh. In the group of L. innocua and L. ivanovii, three samples were of dubious freshness, and in the group of L. monocytogenes, four samples had dubious freshness. Samples of stale meat were not found on the third day of the experiment.

On the fourth day of storage, all samples in the control group remained fresh. In the groups of L. innocua and L. ivanovii, four samples were stale, and in the group of L. monocytogenes, six samples were stale. On the fifth day of the experiment, samples of broiler chicken meat in the groups contaminated with L. innocua, L. ivanovii, and L. monocytogenes were all stale. Six of the samples in the control group of broiler chickens were still good, but one sample was a little stale.

Meat samples were grouped into fresh, dubious fresh, and stale based on the color and transparency of the extract (Table 7). This way, it was easier to figure out what was in the broth.

On the third day of storage of meat by reaction with copper sulfate, test samples from all groups were fresh. On the fourth day, in the control group of broiler chickens, seven samples were fresh, in the groups of L. innocua and L. ivanovii—four samples of stale meat, and in the group of L. monocytogenes—six samples of stale and one sample of fresh meat. On the fifth day of storage in the control group, the meat in six samples was fresh, and only one sample was doubtfully fresh. In other groups of L. innocua, L. ivanovii, and L. monocytogenes, meat samples were stale.

During storage, free fatty acids accumulate in the meat of broiler chickens due to enzymatic hydrolysis. A chemical analysis of the freshness of the fat of broiler chickens is shown in Table 8. Fresh chicken fat has an acid value of up to 1, doubtful fresh fat has an acid value of 1–2.5, and stale fat has an acid value of more than 2.5 mg KOH/gm. Peroxide values of up to 0.01 are allowed in fresh chicken fat, 0.01–0.04 are allowed in dubious fresh fat, and more than 0.04 mg I2/100 gm of stale fat.

Different letters indicate selections that significantly (*p* < 0.05) within the line differ from each other according to the results of the Tukey test, with Bonferroni correction; if there are no letters above the numbers in the line, then no significant difference between any selections is registered.

On the third day of storage of fat samples, the acid value in the experimental groups of L. innocua, L. ivanovii, and L. monocytogenes was higher than the control indicator by 1.8, 2.1, and 2.1 times, respectively. Moreover, a statistical difference was found during the analysis of data from groups L. ivanovii and L. monocytogenes compared with the control indicator (p < 0.05).

On the fourth day of storage of fat samples, the acid value in the experimental groups of L. innocua, L. ivanovii, and L. monocytogenes was greater than the control indicator by 2.1, 2.4, and 2.8 times, respectively (p < 0.05). On the fifth day, the acid value of fat in groups L. innocua, L. ivanovii, and L. monocytogenes was 2.7, 2.6, and 2.7 times higher than in the control group, respectively (p < 0.05).

Regarding the peroxide value of fat on the third day of storage, the indicator of the group of L. ivanovii exceeded the control 1.9 times. The peroxide value of the L. innocua and L. monocytogenes groups was 2.4 and 2.5 times higher than the control indicator (p < 0.05). On the fourth day of storage of fat samples, the peroxide value in the experimental groups of L. innocua, L. ivanovii, and L. monocytogenes was greater than the control parameter by 3.8, 4.1, and 4.9 times, respectively (p < 0.05).

On the fifth day, the peroxide value of fat in the groups of L. innocua, L. ivanovii, and L. monocytogenes was 3.5, 3.4, and 4.3 times higher, respectively, compared with the control group (p < 0.05). Chicken fat from carcasses of the control group by acid and peroxide value was referred to as «fresh» throughout the study period.

On the fourth day of storage, the fat of carcasses of L. innocua broilers was of dubious freshness; on the fifth-stale by acid value; on the third and fourth days of questionable freshness; and on the fifth-stale by peroxide value. The fat of carcasses in groups L. ivanovii and L. monocytogenes by acid and peroxide value on the third and fourth days of storage referred to the dubious freshness, on the fifth-to staleness. Thus, when chicken meat is contaminated with *Listeria*, the fats break down quickly, which leads to the buildup of peroxides and free fatty acids.

## Discussion

Cjurina [23] reports that in experimental listeriosis (L. monocytogenes) at the slaughter of broiler chickens, she found signs of damage to certain organs: hepatic and renal dystrophy, hyperemia of the heart and myocardium, hyperemia of the brain, and edema of the meninges. In our studies, we saw subcutaneous hemorrhage, an enlarged spleen, an overflowing gallbladder, congestive hyperemia of the internal organs, and intestinal vascular hyperplasia when we killed chickens that had been infected.

According to Nikolic et al. [24], the weight of the chilled chicken carcass ranged from 1,628 to 2,414 gm; the breast weight after deboning, from 474.5 to 735.2 gm. According to our research, the weight of carcasses of chickens of all groups (from 1,784 to 1,873) and chicken fillets ranged from 493 to 546 gm. Brewer et al. [25] reduced the carcass yield of chickens from 78.3% to 79%. This coincides with our results: the slaughter yield of broiler chickens of all groups was almost at the same level at 76.4%–77.3%.

Tasoniero et al. [26] received the following breast meat composition: moisture 75.6%–76.9%, protein 20.3%–22.5%, and fat 1.01%–1.8%. In experimental listeriosis, Cjurina [23] obtained the following physicochemical parameters: An infected chicken made up 75.3%–76.2% of the meat, while a healthy chicken made up just over 70% of the meat, fat made up 1.1%–2.2%, and protein made up 19.2%–21%. According to our results, the moisture content in the meat of infected chickens was 74.7%–76.0%, in control—73.2%, the fat content in the infected was 3.3%–4.4%, in control—4.5%, and the protein content in the infected was 20.3%–21.3%, in control—21.5%. Our data coincide with the results of Cjurina [23]: the moisture content of meat in all experimental groups was higher compared to the control group, and a statistical difference was found (p < 0.05).

According to our own data, the water-holding capacity of chicken meat across all groups is noted at a level of 67%–69.5%. Fernandes et al. [27] determined the water-holding capacity of chilled broiler meat at a level of 69.19%. Warner [28] explains that the water-holding capacity of raw muscle changes due to animal genetics, preslaughter stress, antemortem, and post-mortem factors. Tasoniero et al. [26] stated that the greater pH values are typically associated with better water-holding capacity in meat. In their studies, Brewer et al. [25] used a different way to determine how much water chicken can hold, and they used cook loss (22.7%–25.5%).

According to their results, the total plate count of meat of control chickens is 1.4 × 10^2^ CFU/gm, and of chickens infected with *Listeria*, it ranges from 2.6 × 10^2^ to 33.6 × 10^2^. Meat from L. *ivanovii*and L. monocytogenes were contaminated with coliforms. According to Cjurina [23], the total plate count of meat of control chickens is 10–30 CFU/gm. Listeriosis increases the total microbial contamination of meat to 3.6 × 10^2^ CFU/gm, and coliforms are detected in 0.01 gm of meat. Rakhmaev et al. [1] note that the total plate count in broiler meat amounted to 0.5 × 10^4^ CFU/gm.

According to Cjurina [23], the pH of the meat of control broiler chickens after slaughter was 5.9–6.1, and those infected with L. monocytogenes was 6.3–6.4. According to their results, after slaughter, the pH level of the meat did not differ statistically between the control and experimental groups (6.0–6.1). pH 5.84–5.99 in the meat of broiler chickens (first day after slaughter) [26]. According to the results of studies by Bowker and Zhuang [29], the pH on the first day after the slaughter of healthy chickens averaged 5.98–6.17, while the pH of chickens at the time of deboning was 6.30–6.40 [25]. According to Nikolic et al. [24], the pH of chicken was observed to range from 6.19 to 6.27. Wong and Ashton [30] reported a pH of 6.30–6.34 (after slaughter), on the fourth day of chicken storage, 6.42–6.46, and on the fifth, 6.89–6.90. The increase in pH level on the fourth and fifth days of storage coincides with our results: 6.33 ± 0.03 and 6.66 ± 0.07, respectively.

Regarding the analysis of chicken fat, the literature has very limited data. The study by Dhakal et al. [31] evaluated the effects of sodium bisulfate and lactic acid on the shelf-life of rendered chicken fat. Peña-Saldarriaga et al. [32] identified that “the predominant fatty acids in chicken fat by-products were oleic, palmitic, and linoleic acids.” In their works, assessment of fat quality during storage of chicken meat,” they indicate: “Acid number after 12 months of storage was ranged from 5.97 to 8.39 mg KOH/gm of fat; after 15 months, it was ranged from 3.26 to 7.80 mg KOH/gm of fat.” Because of the peculiarities of the method of analysis, the data differ from our research results (acid value after 3 days of storage: 0.55–1.17 mg KOH/gm of fat) [33]. 

## Conclusion

An experimental study showed that the infection with Listeria spp. did not affect the weight of broiler chickens or blood counts, but significantly affected the quality and safety of the meat. At the end of the experiment, in the control group of chickens, all animals (10) were alive; in the groups of L. innocua, L. ivanovii, and L. monocytogenes left 8, 9, and 7 birds, respectively. In some chickens of group L. monocytogenes after slaughter, there were signs of poor bleeding (hemorrhage under the skin) and damage to organs. The fatness of carcasses of L. innocua and L. ivanovii groups was noted, but the presence of hemorrhages in the subcutaneous tissue spoiled the appearance of carcasses. Slaughter yield of broiler chickens of all groups was observed at almost the same level, at 76.4%–77.3%. Experimental infection of broiler chickens with Listeria did not affect the moisture-holding capacity of poultry meat. The moisture content of the meat in all experimental groups was higher compared to the control group (p < 0.05). By bacterial contamination on the 1 day after slaughter, the meat of chickens of the experimental groups L. innocua, L. vanovii, and L. monocytogenes outperforms the control group by almost 1.9, 13.9, and 24.7 times, respectively (p < 0.05). The same trend is observed for the third, fourth, and fifth days of meat storage. Only samples from the control group remained fresh during the normative shelf life of chilled poultry meat (5 days). According to the total bacterial contamination, the meat of broiler chickens with experimental infection with L. innocua and L. *ivanovii* was dangerous to human health after 5 and 4 days of storage, respectively. From the first day after slaughter, the meat of broiler chickens with experimental infection with L. monocytogenes does not meet the requirements (up to 100 CFU/gm). It is not suitable for storage and consumption. According to the content of volatile fatty acids, the meat of poultry infected with L. innocua, L. vanovii, and L. monocytogenes does not correspond to fresh for the fourth day of storage. According to the qualitative reactions to determine the products of the primary breakdown of protein (reaction with CuSO4) and the content of ammonia and ammonium salts, meat in experimental listeriosis remained fresh for no more than 3 days. Listeria contamination of chicken meat contributes to the rapid deterioration of fats.

## List of abbreviations

CFU, colony forming units; gm, grams; ISO, International standardization organization; UNCSM, Ukrainian national collection of strains of microorganisms.
